# Hook tool manufacture in New Caledonian crows: behavioural variation and the influence of raw materials

**DOI:** 10.1186/s12915-015-0204-7

**Published:** 2015-11-18

**Authors:** Barbara C. Klump, Shoko Sugasawa, James J. H. St Clair, Christian Rutz

**Affiliations:** Centre for Biological Diversity, School of Biology, University of St Andrews, Sir Harold Mitchell Building, St Andrews, KY16 9TH UK

**Keywords:** Construction behaviour, Corvid, *Corvus moneduloides*, Extractive foraging, Hook, Material culture, Social learning, Stone tools, Tool manufacture, Tool use

## Abstract

**Background:**

New Caledonian crows use a range of foraging tools, and are the only non-human species known to craft hooks. Based on a small number of observations, their manufacture of hooked stick tools has previously been described as a complex, multi-stage process. Tool behaviour is shaped by genetic predispositions, individual and social learning, and/or ecological influences, but disentangling the relative contributions of these factors remains a major research challenge. The properties of raw materials are an obvious, but largely overlooked, source of variation in tool-manufacture behaviour. We conducted experiments with wild-caught New Caledonian crows, to assess variation in their hooked stick tool making, and to investigate how raw-material properties affect the manufacture process.

**Results:**

In Experiment 1, we showed that New Caledonian crows’ manufacture of hooked stick tools can be much more variable than previously thought (85 tools by 18 subjects), and can involve two newly-discovered behaviours: ‘pulling’ for detaching stems and bending of the tool shaft. Crows’ tool manufactures varied significantly: in the number of different action types employed; in the time spent processing the hook and bending the tool shaft; and in the structure of processing sequences. In Experiment 2, we examined the interaction of crows with raw materials of different properties, using a novel paradigm that enabled us to determine subjects’ rank-ordered preferences (42 tools by 7 subjects). Plant properties influenced: the order in which crows selected stems; whether a hooked tool was manufactured; the time required to release a basic tool; and, possibly, the release technique, the number of behavioural actions, and aspects of processing behaviour. Results from Experiment 2 suggested that at least part of the natural behavioural variation observed in Experiment 1 is due to the effect of raw-material properties.

**Conclusions:**

Our discovery of novel manufacture behaviours indicates a plausible scenario for the evolutionary origins, and gradual refinement, of New Caledonian crows’ hooked stick tool making. Furthermore, our experimental demonstration of a link between raw-material properties and aspects of tool manufacture provides an alternative hypothesis for explaining regional differences in tool behaviours observed in New Caledonian crows, and some primate species.

**Electronic supplementary material:**

The online version of this article (doi:10.1186/s12915-015-0204-7) contains supplementary material, which is available to authorized users.

## Background

New Caledonian (NC) crows (*Corvus moneduloides*) manufacture a diversity of tools, which they use for extracting embedded prey [1, 2]. Two of their tool types have ‘hooks’: pandanus tools (made from the leaf edges of screw pines, *Pandanus* spp.) and hooked stick tools (made from forked plant stems) [1, 2]. While the former have multiple barbs that occur naturally on the plant material (as in the blackberry tools used by woodpecker finches [3]), the latter have a single terminal hook that is actively crafted by the bird [1, 4], representing the only known example of hook production by a non-human species [5]. Hooks were one of the key technological innovations of Middle Stone Age humans (barbed-edged tools *ca.* 90,000 years ago [6, 7]), leading to the development of productive fishing technologies [7–9] and weapons of enhanced killing power [10, 11]. NC crows provide unique opportunities to study the behavioural ecology, and possible evolutionary origins, of basic hook-making skills in a non-human study system.

**Fig. 1:**
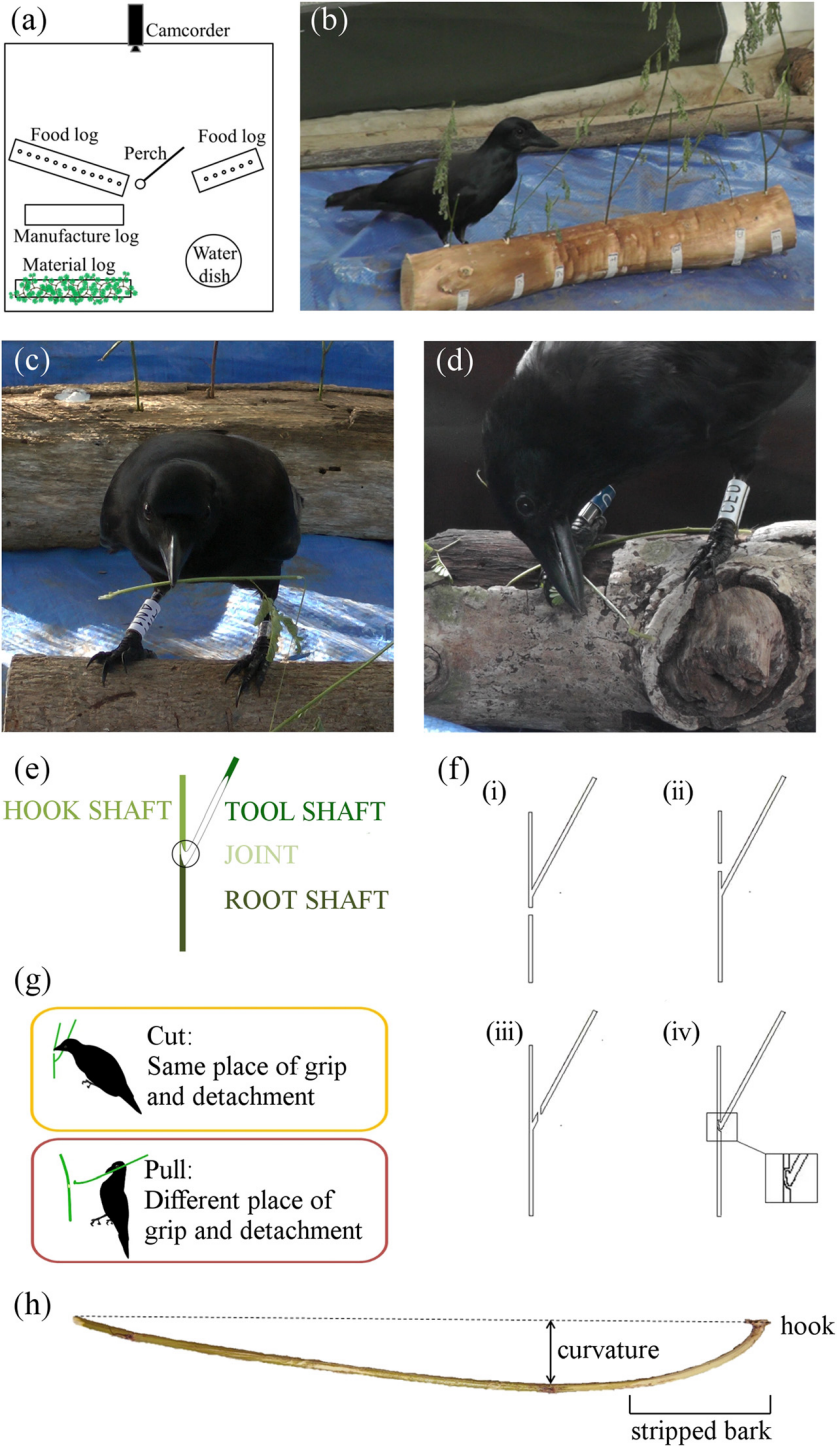
Experimental set-up for investigating hooked stick tool manufacture in New Caledonian crows, and key terminology. **a** Top view of set-up in an experimental chamber (approximately 3 × 3 m), as used in Experiment 1. In Experiment 2, only one ‘food log’ with a single extraction hole was presented. New Caledonian crow **b** examining seven plant stems of different properties in Experiment 2 (presented in randomised order on the ‘material log’), and **c** and **d** processing a basic tool (on ‘manufacture log’ in panel **c**). **e** Schematic drawing of a forked plant stem, and terminology used to score crows’ manufacture actions. The position of the basic tool is highlighted in white. **f** Crows can detach plant material from the stem at the: (*i*) root shaft; (*ii*) hook shaft; (*iii*) tool shaft (results in a non-hooked stick tool); or (*iv*) joint. Note that, by definition, detachment at the tool shaft is only possible through ‘cutting’, and detachment at the joint only through ‘pulling’. **g** Description of the two methods of detaching plant material from the stem. Colour-coding in panels **e** and **g** is the same as in Fig. 4. **h** Photo of a hooked stick tool showing three design features: the crafted hook; stripped bark at the hooked end; and moderate crow-induced curvature of the tool shaft

The manufacture of hooked stick tools in NC crows has previously been described as a complex but highly standardised process, consisting of several basic steps [4]: selection of an appropriate forked plant stem; consecutive removal of material above and below the fork to produce a basic tool; and finally shaping of the hooked end by removing further material (‘crafting’). Given the number of possible behavioural sequences that could potentially produce a hooked stick tool (see Fig. 1e, f), the low level of observed variation seems surprising, but may be due to small sample sizes. So far, only 14 manufacture episodes have been documented: four for unmarked, free-ranging crows [1], and another 10 for two wild subjects that visited a baited feeding table [4].

Behavioural variation within or between populations is the outcome of complex interactions between animals’ genetic predispositions, individual (trial-and-error) and social learning, and/or ecological factors [12]. It has been suggested that, if all mechanisms except social learning can be ruled out, the observed variation can be ascribed to ‘cultural’ processes [13]. Although widely adopted (e.g., [14–17]), this ‘method of exclusion’ to assess putative examples of animal cultures remains highly controversial [18–20]. Importantly, despite good progress with charting genetic and learning effects (e.g., [19, 21–27]), researchers have only recently started investigating the role of ecological factors (e.g., [20, 28–30]). In a pioneering study on chimpanzees’ (*Pan troglodytes*) ant-dipping behaviour, for example, it was found that ecology explained much of the observed between-population differences [28] that had previously been attributed to cultural variation (e.g., [13]). Even though subsequent investigations revealed further subtleties [31] – and it is clear that behavioural variants can be both cultural and locally adaptive [32] – this well-documented case study emphasised that ecological differences can underpin apparent cases of culture to a much greater extent than previously assumed. For NC crows, it has been suggested that geographic variation in the shape of their pandanus tools (see above) is due to cumulative evolution [33, 34], where basic tool designs were progressively refined over time, but evidence for material cultures is still lacking [35].

In tool making [5], and construction behaviour more generally [36, 37], the properties of raw materials are likely to have profound effects on both the behaviour expressed and the morphology of the resulting artefacts – simply by permitting or restricting certain actions – but very few studies have explored these relationships. For humans, the distribution and design of prehistoric tools appear to be correlated with both the quality and quantity of available raw materials [38], although recent experiments revealed that it is possible, in principle, to produce similar stone tools from a range of stone types [39]. Amongst non-human animals, experimentally-provided raw materials have been shown to induce structural differences (compared to wild type) in diverse artefacts, for example, in the protective cases of caddis fly larvae (Trichoptera), when produced from un-preferred raw materials [40], or in the nests of zebra finches (*Taeniopygia guttata*), when built from floppy pieces of string [41]. Given the potentially strong links between raw materials, behaviour and artefacts, and ultimately, their combined effects on fitness components, it is surprising that this topic has received so little attention to date.

To investigate intra- and inter-individual variation in NC crows’ manufacture of hooked stick tools, we provided wild-caught subjects with naturalistic extraction tasks, and multiple stems of the plant material they prefer for tool making (Experiment 1; Fig. 1a). Using a large sample of NC crows, we documented considerable variation in manufacture sequences, as well as novel tool-detachment and processing behaviours. Having observed seasonal change in the abundance and properties of plant materials in our study area, we designed a companion experiment to investigate whether raw-material properties influence behavioural actions during tool production (Experiment 2; Fig. 1b). Taken together, our results show that hooked stick tool manufacture is more variable than previously thought and that the observed variation is at least partly due to plant properties. Apart from providing valuable insights into our crow study system, our findings more generally caution against making premature claims of cultural variation, whilst at the same time highlighting exciting opportunities for future studies of the effects of raw materials on animal construction behaviour.

## Results

### Experiment 1 – Variation in tool-manufacture behaviour

**Fig. 2:**
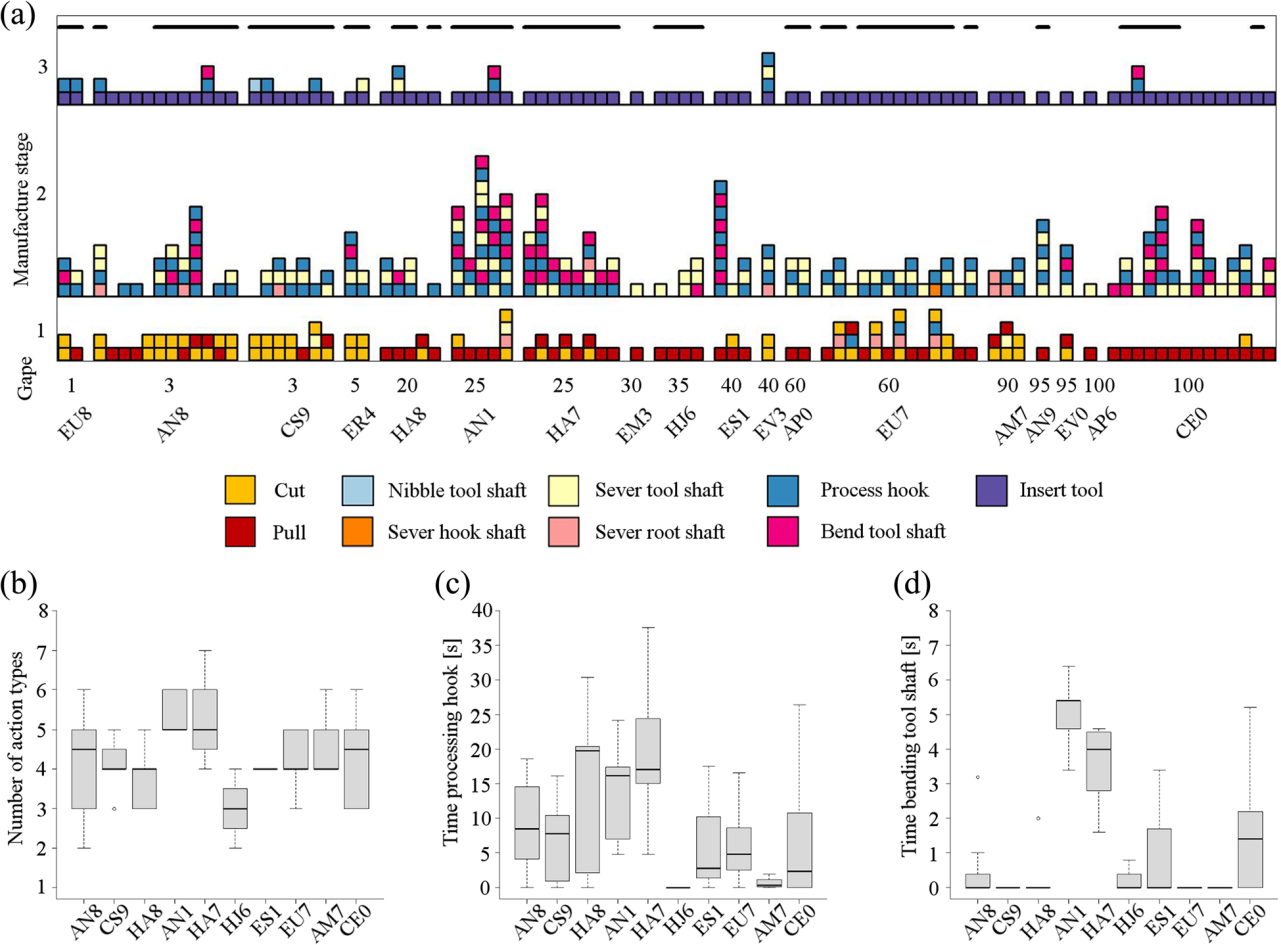
New Caledonian crows’ manufacture of hooked stick tools in Experiment 1. **a** Stacked bars are 85 sequences of behavioural actions from 18 subjects. Actions are colour-coded, and grouped into three main stages: (1) release of the basic tool; (2) processing of the basic tool; and (3) deployment of the tool. Note that, when the subject first detaches the raw material at the hook shaft or the root shaft, it can carry out other actions before releasing the basic tool at the root shaft, the hook shaft, or the joint (Fig. 1f). Sequences are ordered from left to right according to subjects’ gape colouration (% black; older birds tend to have darker gapes – for details, see Methods) and identity (multiple sequences from the same bird are grouped together). Sequences during which crows stripped bark are marked with *black bars* at the top. For manufacture sequences of Experiment 2, see Additional file 6: Figure S1. **b**, **c** and **d** show, respectively, the number of action types per sequence, the time spent processing the hook, and the time spent bending the tool shaft, for 10 subjects that had each produced three or more sequences. Boxplots show, where applicable, the median (*thick line*), the first and third quartiles (*lower and upper margins of box*), an approximate 95 % confidence interval (*whiskers*), and outliers (*empty circles*)

Each crow was provided with an extraction task and several forked stems of *Desmanthus virgatus* (Fig. 1a) that were judged to be suitable for hooked stick tool manufacture (based on tools recovered from free-ranging crows [42]). We distinguished two methods of releasing a ‘basic tool’, by scoring the place where the subject gripped the plant material (root shaft, hook shaft, tool shaft, or joint; Fig. 1e) and the place of subsequent detachment (Fig. 1f): if these were identical, the behavioural action was considered a ‘cut’, and otherwise a ‘pull’ (Fig. 1g). Releasing a basic tool from a forked stem thus requires two cuts, a cut and a pull, or a single pull. While the cut method had been observed before [1, 4], we report here the first conclusive documentation of techniques involving pulls, which are commonly employed by crows in our study population (16 of 18 subjects; see Fig. 2a and Additional file 1: Movie 1).

After the release of the basic tool, crows used seven different processing action types (Fig. 2; for definitions, see Additional file 2: Table S1), including bark stripping and bending of the tool shaft. Following an initial observation that tools recovered in our study site often lacked bark at the hooked end and exhibited pronounced curvature [43] (see Fig. 1h), we confirmed with our behavioural experiments that these design features are actively induced by crows (Additional file 3: Movie 2), rather than merely being a by-product of tool detachment or deployment. Although we did not score bark stripping systematically, since it often coincided with the processing of the hook, 12 of our 18 (67 %) subjects unambiguously expressed the behaviour at least once during experimental trials (Fig. 2a). Ten of our 18 subjects (56 %) exhibited tool-bending behaviour, which involved: (i) trapping the tool under one or both feet and bending the tool shaft by bill (45 times by 10 subjects); (ii) holding the tool in the bill and pressing the tool shaft or tool tip against a firm substrate, such as the floor or a log (8 times by AN1); or (iii) inserting one end of the tool into a hole and pulling the tool shaft sideways by bill (3 times by HA8, outside of formal scoring) (Additional file 3: Movie 2, Scenes 2–4). The majority of tools produced during trials were noticeably curved (Fig. 1h), but it remains to be quantified how much extra curvature crows manage to add to stems during tool processing.

Individuals differed significantly in the median: number of action types per manufacture sequence (Kruskal − Wallis test [KWT]: *χ*^2^_9_ = 19.94, *P* = 0.018; Fig. 2b); time spent processing the hook (KWT: *χ*^2^_9_ = 24.82, *P* = 0.003; Fig. 2c); and time spent bending the tool shaft (KWT: *χ*^2^_9_ = 48.13, *P* <0.001; Fig. 2d). None of these measures was significantly related to gape colouration (a proxy of bird age – see Methods; generalised linear mixed models [GLMMs]: *χ*^2^_1_ = 0.004–1.35, *P* = 0.24–0.95). Sequences of behavioural actions during processing varied significantly more between subjects, than within subjects (permutational ANOVA [PA]: *R*^*2*^ = 0.512, *P* = 0.027), but differences were not correlated with gape colouration (Mantel test [MT]: *R* = −0.132, *P* = 0.94).

There was some variation in how consistently individuals expressed key behaviours (Fig. 2a). For instance, HA7 and EU7 used both cut and pull as their first actions interchangeably, while CE0 (see Additional file 4: Movie 5) and CS9 (almost) exclusively used pulls and cuts, respectively. Similarly, AN8 only sometimes bent the tool shaft, in contrast to AN1 and HA7 (see Additional file 5: Movie 4) who bent in every sequence, and EU7 and AM7 who never exhibited the behaviour. Finally, some birds – such as AN8, HA8 and CE0 – stripped bark occasionally, while others were more consistent: AN1 and HJ6 always peeled bark, and ES1 and AM7 never did. Since birds were kept in aviaries for comparatively brief periods of time, some of these seemingly idiosyncratic differences may have been due to seasonally changing raw-material properties, as explored in detail below.

### Experiment 2 – Effects of raw material on tool-manufacture behaviour

**Fig. 3:**
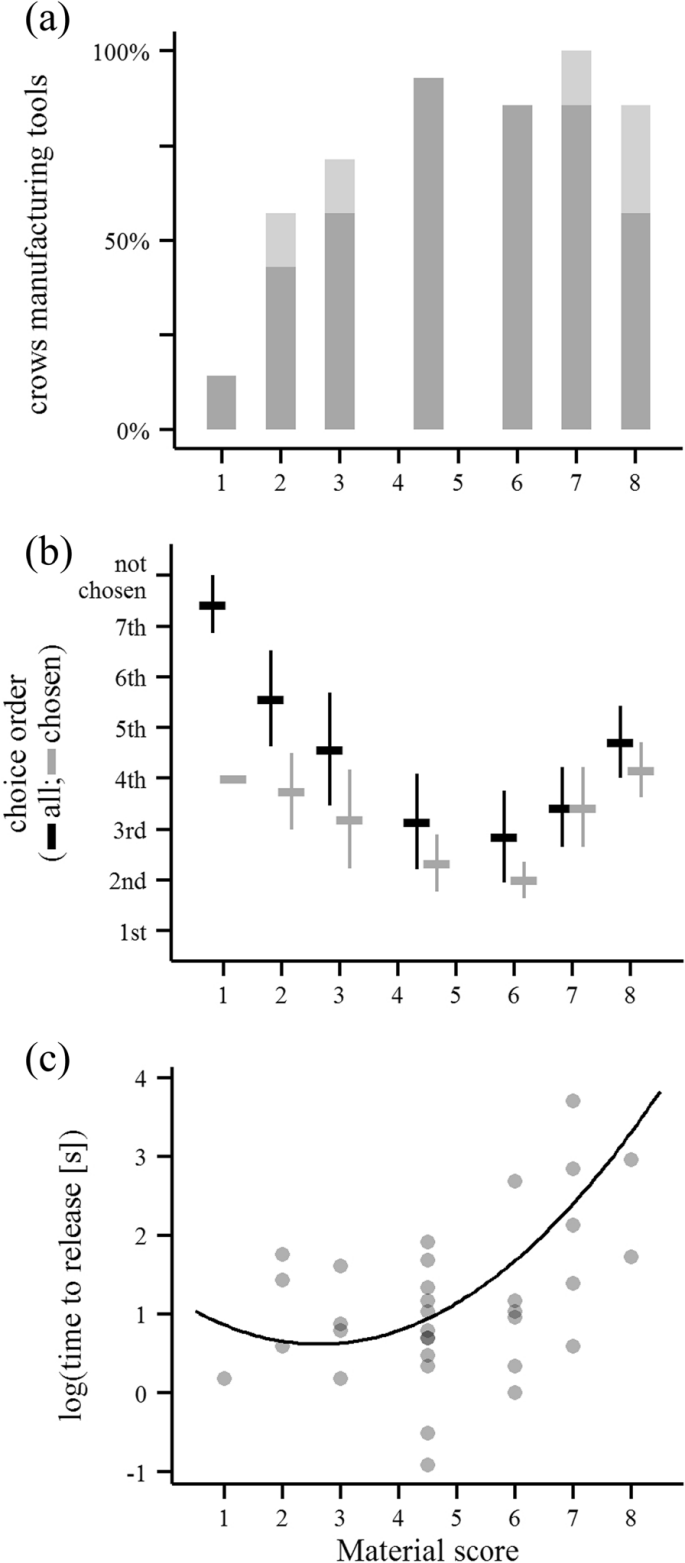
Influence of raw-material properties on the tool-manufacture behaviour of New Caledonian crows in Experiment 2. **a** Percentage of crows manufacturing tools from the stems provided in choice trials (*n* = 14 for material score 4.5 [‘matched’ plus ‘control’ stems]; *n* = 7 for all other material scores; *dark grey*: hooked stick tools, *light grey*: non-hooked stick tools). **b** Order (mean ± standard error) in which stems were chosen by crows (excluding control stems, but including stems which were pulled out of the log or used to make non-hooked stick tools). *Black values* are for all stems, with unchosen stems being assigned a value of 8 (*n* = 7 for each material score), and *grey values* are for chosen stems only (*n* = 1–7). **c** Time to release the basic tool (only hooked stick tools, with stems that were pulled out of the log excluded; *n* = 1–12)

Seven subjects were each provided with eight plant stems of different properties (see Fig. 1b), ranging from green and flexible (‘material score’ 1) to woody and rigid (score 8), with the two stems in the middle of the range (scores 4 and 5; ‘control’ and ‘matched’ stems) matched as closely as possible (for details, see Methods). Initially, only seven of these stems were presented for the crow to choose from, with the control stem held in reserve (see below). When a bird had chosen one of these seven stems to manufacture a tool and extract bait, this tool was removed and the crow could choose again from the remaining stems; at the end of the trial the control stem was presented. All birds manufactured tools from – and extracted bait with – the control stem, confirming that non-manufacture of tools from any of the seven earlier stems reflected the subject’s assessment of plant properties, rather than resulting from satiation or demotivation. Taken together, the subjects of this experiment manufactured 42 tools (4–8 per bird) out of the 56 provided stems (Fig. 3a). Thirty-seven of the manufactured tools were hooked stick tools, and five were non-hooked stick tools.

We recorded the order (choice 1–7) in which crows chose the provided stems (control stems were not considered in this analysis, as they were always presented last; see above). Stems that were not used by subjects were assigned a value of 8. Using a permutation test [44], we found that subjects chose stems of material score 6 significantly earlier than expected by chance (*P* = 0.035) and tended to do so for stems of material scores 4 and 5 (*P* = 0.097; Fig. 3b). Controlling for individual differences (bird ID fitted as a random effect), material score predicted whether or not crows manufactured a hooked stick tool out of a stem (GLMM: *χ*^2^_2_ = 21.57, *P* <0.001), with fewer hooked stick tools produced from stems at either end of the presented range (Fig. 3a). Crows spent least time gripping a stem before releasing the basic tool (a proxy of manufacturing effort) when handling stems of material score 3 (GLMM: *χ*^2^_2_ = 10.72, *P* = 0.005; Fig. 3c).

**Fig. 4:**
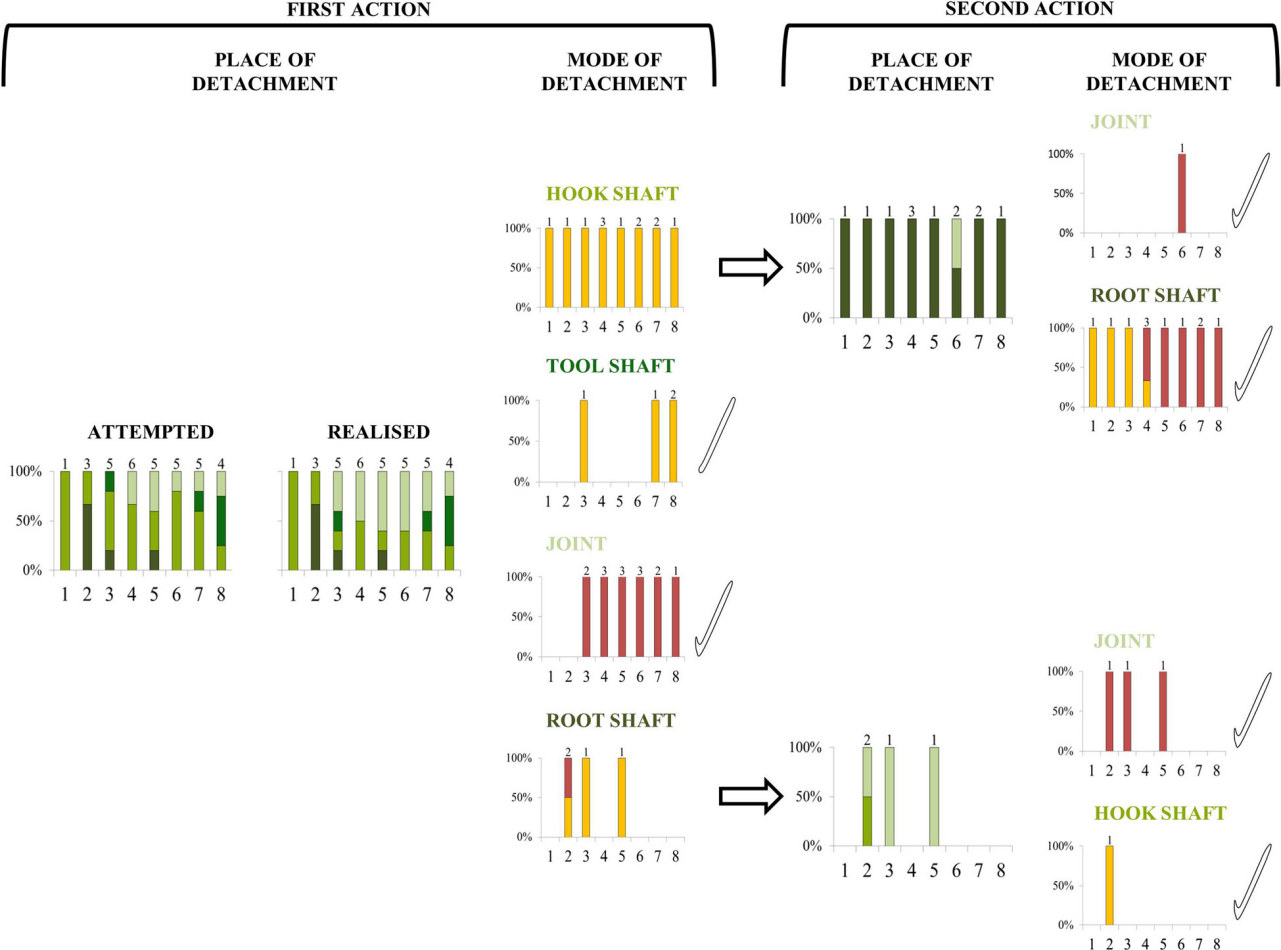
New Caledonian crows’ method of releasing basic tools in Experiment 2. Place and mode of detachment for the first (*left*) and second (*right*) actions of manufacture sequences; for a detailed explanation of colour-coding and terminology, see Fig. 1. The attempted and realised place of detachment is only shown for first actions, as they were the same for all second actions. Tool icons indicate when the basic tool (hooked or non-hooked) was released from the plant stem, and numbers above bars are manufacture sample sizes across subjects. Three manufacture sequences (all by bird EV0) are not shown, as they deviated from the general pattern (two incomplete detachments at the hook shaft with subsequent detachment at the root shaft, and one detachment at the hook shaft followed by a detachment at the root shaft, which was clearly a cut action)

Sequences tended to contain more actions when the stems’ material score was high (GLMM: *χ*^2^_1_ = 3.69, *P* = 0.06). Pulls appeared to be used more frequently with higher material scores (Additional file 6: Figure S1), but this relationship was non-significant (GLMM: *χ*^2^_1_ = 2.44, *P* = 0.12). Given the multitude of possible release strategies (see different ‘pathways’ from left to right in Fig. 4), it is unsurprising that this global statistical model failed to pick up an effect of raw-material properties. Interestingly, however, closer inspection of the data revealed a very consistent pattern in a subset of cases: if the first detachment happened at the hook shaft, the second action was always a cut for stems of material scores 1–3, and a pull for stems of scores 5–8 (Fig. 4). Of the five birds with valid manufacture sequences for both matched and control stems, three used the same first action (place of grip and place of detachment) for both stems, one attempted to do so (incomplete action for control stem), and one bird used different first action types. This sample is insufficient for formal statistical analyses, but the repeatability of results is qualitatively consistent with an effect of raw materials on manufacture behaviour.

### Seasonal patterns of tool-manufacture behaviour

The availability and properties of the crows’ preferred plant materials change significantly in our study site over the course of a few months, from August onwards, as the rainy season is approaching (van der Wal et al. unpublished observations). Initially, young growing stems are relatively thin in diameter and flexible, while later on, stems will get thicker in diameter and/or become woodier. Although we attempted to standardise the stems used for Experiment 1, to correspond to the tools we had previously recovered from free-ranging crows (and which accord with the preferred middle range of material scores in Experiment 2), our selection was inevitably subject to environmental availability. Given that raw material influenced hooked stick tool manufacture in Experiment 2, we investigated the possibility that temporal changes in raw materials might have driven at least part of the variation observed in Experiment 1.

**Fig. 5:**
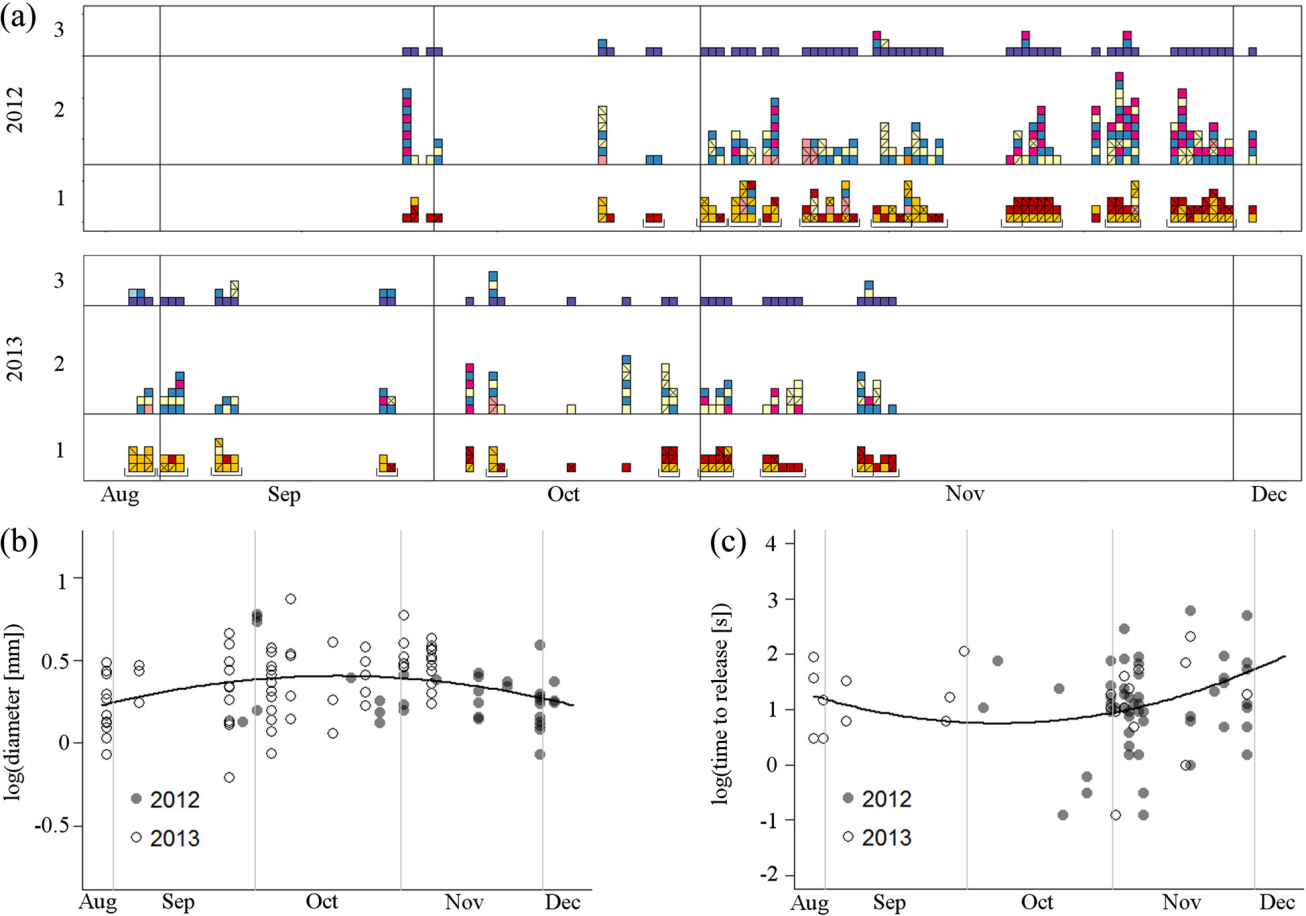
Seasonal variation in New Caledonian crows’ tool-manufacture behaviour. **a** Manufacture sequences from Experiment 1 (as shown in Fig. 2a) reordered by calendar date, and with information added on incomplete behavioural actions and actions that were completed through multiple attempts. For a description of colour coding and manufacture stages, see Fig. 2. Sequences produced on the same day are grouped with a bracket underneath. Most-effective attempts and incomplete attempts are marked (/) and last attempts are marked (\); note that (/) and (\) can coincide (×). Temporal change in **b** the diameter of manufactured tools, and **c** the time taken to release the basic tool, with corresponding model fits (for further details, see main text)

The diameter of stems used by crows in trials of Experiment 1 changed (non-significantly) over the course of the season (linear mixed model [LMM]: *χ*^2^_2_ = 5.75, *P* = 0.06), peaking in October (Fig. 5b). While this change in itself is not very informative, as it is due to the combined effects of our sampling of raw materials and crows’ choices (rather than reflecting the environmental availability of materials *per se*), it is noteworthy that the pattern was mirrored by our estimate of manufacture effort, which was lowest in October and increased afterwards (LMM: *χ*^2^_2_ = 8.23, *P* = 0.02; Fig. 5c). In fact, in Experiment 2, the stems that crows detached most quickly (i.e., the stems of material score 3) were of comparable diameter to early-October tools in Experiment 1 (Additional file 7: Figure S2), providing further support for a link between material properties and crow behaviour.

In order to examine possible seasonal changes in hook tool manufacture sequences, we re-plotted all sequences from Experiment 1 in temporal order, this time including incomplete behavioural actions and actions that were completed through multiple attempts, to highlight cases where birds may have struggled with the material (Fig. 5a). The number of actions in manufacture sequences increased (non-significantly) over time (generalised linear model [GLM]: *χ*^2^_1_ = 3.59, *P* = 0.06), and processing sequences became significantly more dissimilar to each other the more time had passed between trials (MT: *R* = 0.07, *P* = 0.03). For reference, in Experiment 2, processing sequences tended to be more dissimilar for stems of divergent material scores (MT: *R* = 0.16, *P* = 0.07).

## Discussion

We conducted the largest-ever experimental study on NC crows, to elucidate aspects of the species’ intriguing hook-making behaviour. Experiment 1 revealed high levels of within- and between-individual variation in the manufacture sequences of hooked stick tools, including hitherto-undescribed behaviours. Experiment 2 demonstrated that plant properties significantly influenced the manufacture process, and complementary analyses suggested that at least part of the behavioural variation observed in Experiment 1 can be explained by seasonally-changing raw materials.

The release of the basic tool is an important step in the manufacturing process of hooked stick tools, since it results in a functional tool, even though crows will usually continue with further processing. In contrast to an earlier study [4], where two crows were observed to release basic tools from the plant by cutting the hook shaft, and then cutting the root shaft, our subjects released basic tools also by a combination of cutting and pulling actions, or simply by pulling the tool shaft (see pathways in Fig. 4). The latter technique enables crows to manufacture a functional, albeit crude, hooked stick tool with a single behavioural action. Based on first glimpses of manufactures in the wild, Hunt [1] inferred that crows may use a single ‘nipping cut’ for detaching a basic tool, but he later appeared doubtful of this interpretation [4]. It is impossible to tell whether these were behaviours that we would now class as detachments at the joint using a single ‘pull’, but some of our subjects clearly gripped tool shafts some distance away from the joint when pulling, which has not been previously described. It is also interesting to note that we observed crows making distinctly hooked tools with a single swift pull action, and deploying them straightaway, without any hook processing (see Fig. 2 and Additional file 6: Figure S1), which contrasts with Hunt’s [1] earlier observations. We suggest that such a one-step approach is a likely precursor of multi-step manufacture techniques, such as ‘cut–pull’ or ‘cut–cut’. These more involved techniques would not only afford more control over the eventual shape of hooks, but they would also permit hooks to be produced from plant species, or stems with certain material properties, where a simple pull might not work [4].

If hooked tools are more efficient than non-hooked variants for foraging, we would expect selection to favour their production by crows, and potentially, even their progressive technological refinement [33, 45, 46]. Apart from the hook itself, hooked stick tools produced by NC crows in our study site have two additional design features that are likely to increase foraging efficiency (Fig. 1h) [43]: a curved tool shaft (which may help birds position the hooked end of the tool in the centre of their field of binocular vision, enhancing precise handling [43, 47]), and a bark-free functional end (which may improve the visibility of the tool in dark cavities, and could reduce mechanical resistance during probing [43]). Our experiments provided the first detailed documentation of how NC crows induce these features during the manufacture process (for an earlier mention of bark stripping, see [1]). Given that they are not necessary, but may significantly improve tool functionality, bending and bark stripping may have been (cumulative) additions to the hooked stick tool manufacture repertoire, following the discovery of the initial detachment step.

Natural variation in behaviour can be generated by a range of factors, including differences in genetic make-up, individual and social learning, and/or ecological conditions [12]. The absence of age-dependent changes in tool-manufacture behaviour (Fig. 2) was surprising, since NC crows have been previously reported to hone their tool-related skills during a protracted developmental period [48–50]. Notwithstanding some uncertainty in the ageing of corvids based on gape colouration (see Methods), we are confident that birds with black gapes (>90 % black) were adults – a group of subjects that was found in our experiments to exhibit considerable variation in manufacturing behaviour (Fig. 2). At the other extreme, some subjects in our sample were evidently still very young, based both on their physical appearance and behaviour (e.g., persistent begging by EU8), yet exhibited highly dexterous tool-manufacture behaviour (see Additional file 8: Movie 3). This could indicate either the existence of specific genetic predispositions [23, 51], or that crows learn the required manufacture techniques very early on during development. It seems likely that individual and social learning contribute to the acquisition of manufacture techniques, but further work is required to quantify such effects.

The properties of raw materials affected several aspects of NC crows’ manufacture behaviour. Although subjects were able to manufacture tools from material that varied substantially in robustness, they exhibited a clear preference for stems in the mid- to high-end of the provided range (Fig. 3a). Manufacture from stems of either extreme of the provided spectrum proved difficult, and we had the impression (based on our observation of subjects engaging with these materials during trials, and close examination of tools and plant debris after trials) that tools produced from very thin and green material were generally too flexible or fragile to be functional. Exactly how raw-material properties influence the morphology, and efficiency, of the resulting tools, remains to be investigated, but our study cautions that plant materials may contribute to between-population variation in NC crow behaviour [20, 28–30]. Thus, when assessing the possibility of material culture in these birds [33, 34], it is essential to chart the availability and usage of different plant species [42] (for a recent study on chimpanzees, see [52]), as well as their mechanical properties.

Plants change seasonally, as rainfall and rising temperatures promote growth, flowering and fruiting. Indeed, we found in our study site that the abundance and appearance of plant stems used by crows for tool manufacture changes considerably over relatively short time periods (van der Wal et al. unpublished observations). Despite our efforts to standardise the materials presented to crows in Experiment 1, the diameter of tools changed as the season progressed, peaking in October (Fig. 5b). A concurrent change in manufacture effort (Fig. 5c) suggested that crows found it easiest to handle stems of a diameter that matched the ‘optimal’ range identified by Experiment 2 (Additional file 7: Figure S2). Furthermore, in Experiment 1, sequences became more divergent the more time had passed between trials, which again appears to agree with Experiment 2, where sequence dissimilarity was a function of material properties. On the other hand, based on results from Experiment 2, we would have expected sequence length in Experiment 1 to track changes in stem/tool diameter (as shown in Fig. 5b), resulting in shorter sequences towards the end of the study period – instead, we observed a progressive increase in sequence length (Fig. 5a), which would imply that material properties other than diameter had changed. This said, temporal patterns shown in Fig. 5 should be treated cautiously, as we had to pool data from two study years (2012 and 2013), which may have differed in plant phenology and/or fieldworkers’ selection of experimental stems. Although it is admittedly difficult at present to reconcile all available pieces of evidence, our analyses provide important first pointers that changes in raw materials may influence NC crows’ hook tool manufacture behaviour. Future studies must now take on the challenge of experimentally isolating the effects of purely dimensional and mechanical plant properties, and of systematically charting seasonal changes in raw materials. This topic is of wider significance, as such seasonality effects are likely to play a role for a range of other animal construction behaviours, most notably avian nest building [36], as indicated by first data for two weaver bird species [53].

A number of studies have investigated how animals choose raw materials for their constructions [36], including tool manufacture [5]. Such selectivity is crucial, as the properties of the raw material may influence subsequent behaviour, and the morphology of the resulting artefact, simply by requiring, allowing or preventing certain actions. Wild bearded capuchin monkeys (*Sapajus libidinosus*) select materials that allow them to crack nuts efficiently [54]; chimpanzees prefer particular tree species for the construction of their night nests [55], and manufacture different tool types from different plants [56, 57]; and orang-utans (*Pongo abelii*) choose rigid branches for building the main structure of their nests, and thinner ones of the same tree for lining them [58]. All these examples suggest that, for certain tasks, some materials are better than others, presumably because of their properties and the way they can be handled. But, they do not answer the crucial question of how the material itself influences construction behaviour, and the morphology of the resulting artefact.

Although this topic has been largely overlooked in the literature on non-human animals, the influence of raw materials on the morphology of human stone-tool artefacts has been discussed for over a century [39]. Interestingly, experimental studies have reached different conclusions about the degree to which variation in artefacts can be attributed to raw-material constraints. Raw material had a predictable influence on flake-breakage patterns during knapping, which highlights the fact that any attempt to use these patterns to infer past human behaviour should control for material properties [59]. On the other hand, a modern stone-knapper was able to produce morphologically indistinguishable tools from a range of raw materials, which emphasises the danger of assuming that differences in raw materials between sites can entirely account for variation in tool morphology [39]. Both raw-material constraints and the manufacturer’s technical competence (manual dexterity or skill) can mediate the influence of raw material on tool manufacture. Whereas archaeologists are confined to investigating the manufacture process and material properties separately – either by observing modern humans creating replica tool artefacts from different materials, or by searching for associations between the morphology of pre-historic artefacts and the environmental distribution of raw materials – non-human study systems provide unique opportunities to study the complex interactions between material properties, manufacturing behaviour, and the morphology of the resulting tools.

## Conclusions

Our discovery of novel tool manufacture behaviours in NC crows enabled us to construct a plausible scenario for the evolutionary origins, and gradual refinement, of the species’ remarkable hooked stick tool making. Furthermore, our experimental demonstration of a link between raw-material properties and aspects of tool manufacture provides an alternative hypothesis for some of the regional differences in tool behaviour observed in NC crows, and a range of primate species. While many earlier studies have treated possible ecological correlates of animal tool behaviour as inconvenient confounds, our study highlights opportunities for exciting future research in this area [2, 20, 28, 60]. Our experimental approach can be applied, in principle, to any animal species that is known to manufacture tools, nests, or other artefacts [5, 36], providing scope for broad taxonomic comparisons.

## Methods

### Study site, subjects and housing

From 17 September to 28 November 2012, and from 24 August to 28 October 2013, we non-selectively trapped NC crows with meat-baited whoosh nets in our farmland study site in Gouaro-Déva, on the central west coast of New Caledonia, South Pacific. Birds were sexed based on morphology (males are larger than females [61]) and aged based on gape colouration (as in other corvids, gape colour in NC crows changes over time from pink, through mottled grey, to black [49, 62]). Two of the subjects (CS9 and ER4) were trapped in two consecutive years (2012 and 2013) and retained a similar level of gape colouration, which indicates some uncertainty in the ageing method (which may be due to social dominance effects – see [62]). This said, gape colouration provides a useful proxy of the general developmental stage of subjects, and enables identification of the youngest and oldest birds in a sample [63]. Crows were housed individually in field aviaries (3 × 3 × 2.5 m) with the exception of adults that had been trapped with dependent young, which were always kept together.

The tool behaviour of 34 crows was assessed in pre-testing sessions (for details, see [43]), and only birds that were confirmed to manufacture and use hooked stick tools progressed to the main experiments [43, 63]. Twenty-nine crows were tested in Experiment 1 (18 in 2012, 14 in 2013; three crows participated in both years), with seven of them also participating in Experiment 2 (all in 2012). Subjects were tested individually in an experimental chamber (connected to the housing aviary), which had opaque side walls to ensure that they could not see, and were themselves not visible to, any other crows during formal trials. To facilitate motivation, food bowls were removed from the housing aviary *ca.* 1–1.5 hours before trials of Experiment 2, and some trials of Experiment 1. During experimental trials, birds had *ad libitum* access to water, but not to food except for the bait provided in extraction tasks. Observers filmed crow behaviour with a Panasonic HD camcorder for subsequent analyses, from a hide outside the experimental chamber (Fig. 1a).

### Experiment 1

#### Experimental procedures

The basic experimental set-up is schematically illustrated in Fig. 1a. We presented raw materials for tool manufacture on one or two ‘material logs’, by firmly wedging stems of *Desmanthus virgatus* (3–12 stems, each usually containing multiple forks) into drilled holes to stand upright, as crows would encounter them in the wild [42]. Up to two ‘food logs’ contained between 6 and 18 drilled holes each (diameter either *ca*. 9 mm or 12 mm; depth 70 mm), which were baited with a peanut-sized piece of pork or beef heart, or a dead spider. In some trials, a ‘manufacture log’, one part of a split wooden log, was presented between the material and food logs, to provide additional surfaces for birds to craft their tools on (Fig. 1a, c). Trials lasted for 90 minutes, but finished earlier if the subject had extracted all bait.

#### Data analyses

For each tool-manufacture sequence, we scored the behavioural actions of three stages (Fig. 2a): the release of the ‘basic tool’ (Fig. 1e); the processing of the basic tool; and the deployment of the ‘tool’ (once the basic tool was inserted into a hole, it was considered a tool). We scored behaviours until the subject extracted bait from a hole, abandoned the tool (i.e., did not touch it with its bill or feet for more than two minutes), or five minutes had elapsed after the first tool insertion into a hole. Videos were scored with JWatcher (www.jwatcher.ucla.edu) and Solomon Coder (www.solomoncoder.com) software, using the definitions provided in Additional file 2: Table S1; note that, unlike an earlier study [4], we did not score removal of leaves or small side branches, as these actions can only be expressed when plant stems possess these structures, which would lead to unreliable estimates of behavioural variability. Inter-observer agreement was assessed as described below^1^.

To enable meaningful comparisons within and between birds, manufacture sequences were excluded if: the length of any of the shafts of the provided plant material was shorter than *ca.* 20 mm; there were more than two branches growing out of, or within *ca.* 20 mm from, the chosen joint; the subject abandoned the plant material or basic tool before its insertion; it pulled out an entire plant stem from the material log; it made a tool out of plant debris (from previous manufactures); or it made a tool by cutting the tool shaft (which results in a non-hooked stick tool; see Fig. 1fiii). Of the 29 crows which participated in Experiment 1, 18 produced at least one manufacture sequence that met these criteria, yielding a total sample of 85 valid sequences.

Using non-parametric Kruskal − Wallis tests, we analysed between-bird variation in (Fig. 2b–d): (i) the number of different action types; (ii) the time spent processing the hook; and (iii) the time spent bending the tool shaft (for definitions, see Additional file 2: Table S1). Subjects that had produced fewer than three valid sequences were excluded from these analyses, leaving a subsample of 74 sequences from 10 subjects. To assess the effect of gape colouration on (i), we ran generalised linear mixed models (GLMMs) using the ‘lme4’ package [64] in R [65], with a Poisson error structure and log link function, and with bird ID fitted as a random effect to account for data non-independence. For all GLMMs, generalised linear models (GLMs) and linear mixed models (LMMs), significance of main effects was assessed with likelihood-ratio tests (best model against null model, at *α =* 0.05). Since metrics (ii) and (iii) were not normally distributed and included zero values, we analysed these data in two steps: we first used a GLMM with a binomial error structure and logit link function to test whether gape colouration was related to the expression of the behaviour of interest (yes/no score). For the sample of sequences that included the behaviour, we then specified a second model with a gamma error structure and inverse link function, to test the influence of crow ‘age’ on the time spent performing the behaviour.

We additionally examined the similarity between manufacture sequences using Needleman − Wunsch distance (NW distance), a measure commonly used in genetic analyses [66]. This method first aligns two sequences (in our case, of behavioural actions during the ‘processing’ stage of tool manufacture where path dependence was assumed to be negligible; see Fig. 2a) so that the number of differences is minimised, and then counts this number of differences; if two paired sequences differ in length, actions missing in one of them are treated as ‘deletions’ in the other. In order to avoid overrepresentation of subjects with more sequences, we picked the first three sequences from each subject and calculated the NW distance for each pair (30 sequences for 10 subjects) using the ‘Biostrings’ package [67] in R. We tested whether NW distance was correlated with individual ID (by multivariate permutational ANOVA), or gape colouration (by Mantel test), using the ‘vegan’ package [68] in R.

### Experiment 2

#### Experimental procedures

To investigate the effects of raw-material properties on tool-manufacture behaviour, we presented subjects with plant stems that encompassed the full natural range of plant properties. In this experiment, we ensured that stems contained only a single fork suitable for hooked stick tool manufacture, removing side branches where necessary (Fig. 1b). When preparing trials, three people (always including BK), who had previously observed hook tool manufacture by captive crows, independently used their best judgement to assign ‘material scores’ to eight stems. Specifically, they were briefed to assess the difficulty that they believed a crow would experience when attempting to sever each stem immediately below the joint on the root shaft. This method exploits the fact that, in addition to assessing basic dimensional properties (such as thickness), human observers can evaluate biomechanical characteristics (such as rigidity) that would be difficult or impossible to measure non-destructively. Material scores ranged from 1 (green and flexible) to 8 (woody and rigid). The median of the three independent scores for each stem was used to assign a final score (inter-observer agreement was excellent^1^). Materials were selected so that the two stems in the middle of the range (material scores 4 and 5) resembled those preferred by crows in the wild [42], and matched each other as closely as possible (for formal analyses both were given the same material score). One of these matched stems was randomly selected to serve as a ‘control’, allowing us to test for demotivation of birds at the end of a trial (see below). The other seven stems were presented simultaneously, arranged side-by-side in random order, on a material log (Fig. 1b).

We provided a food log with a single drilled hole (diameter 16 mm; depth 70 mm), baited with a peanut-sized piece of pork or beef heart, and in some trials, a manufacture log (see above). Before the bird entered the experimental chamber, a tiny piece of meat was positioned on the food log next to the drilled hole to attract the subject’s attention. After each tool manufacture with successful bait extraction, the observer called an assistant by radio. The assistant removed the tool and any plant debris (but left the remaining plant stems in place for subsequent choices), and re-baited the food log, in full view of the subject. If a bird manufactured a tool but did not extract any bait with it within 15 minutes, the tool and plant debris were removed, but the remaining plant stems stayed on the material log, and the food log was re-baited. After all choices had been made, or 15 minutes passed without a tool manufacture, any remaining stems were removed and the control stem was presented on the material log in the position where the matched stem had been presented previously, and the food log was re-baited. The trial finished when the subject used this control stem to manufacture a tool and extract bait.

#### Data analyses

Videos were scored as described above for Experiment 1, and results of inter-observer agreement evaluations are reported below^1^. In Experiment 2, we used more relaxed exclusion criteria for manufacture sequences than in Experiment 1, since provided materials were more rigorously controlled, and the experiment was specifically designed to tempt crows to use non-preferred plant materials. No stems were excluded from our analyses of basic manufacture decisions (56 stems; Fig. 3a), and of the order in which stems were chosen (56 stems; Fig. 3b). Only sequences in which the subject either manufactured a non-hooked stick tool (five cases), or pulled out an entire plant stem from the material log (four cases), were excluded from further analyses, as this inevitably restricted the range of subsequent behavioural options. The final dataset comprised of 33 manufacture sequences (see Additional file 6: Figure S1).

The choice of a stem was scored when the basic tool was released (Fig. 1e), even if a bird had previously interacted with one or more other stems without releasing a basic tool. Subjects’ preferences for stems of particular material scores were analysed using a custom-written permutation test. Given the actual number of choices made by each subject during experimental trials, we performed 10,000 permutations to calculate the mean choice order of any candidate stem under the assumption of random choice (null hypothesis), and then compared the observed choice with this random distribution. We also scored how birds released basic tools from stems (Fig. 1g), as we expected material properties to be particularly important at this early stage of the manufacture process. Given the range of possible pathways that can lead to the release of a basic tool (Fig. 4), and modest replication for some of these behavioural sequences, we used the following rule to group cases for analyses: if the first and second actions were both cuts, the release method was scored as ‘cut’, while if any action was a pull, the release method was considered to be a ‘pull’ (for definitions, see Fig. 1g). Using frame-by-frame analysis, the time required to release the basic tool was measured to the nearest 0.2 seconds.

We used (G)LMMs, with bird ID fitted as a random effect (see above), to analyse the effect of material score on: (i) whether or not a hooked stick tool was manufactured; (ii) the time taken to release the basic tool (log-transformed to normalise errors); (iii) the number of behavioural actions in each sequence; and (iv) the method chosen for releasing the basic tool. For (i) and (ii), we fitted a quadratic term in our models, as birds seemed to struggle with stems at either end of the range, and models including this term had lower AIC values than those without (for [i]: 56.20 vs. 67.66, *P <*0.001; for [ii]: 90.88 vs. 92.13, *P =* 0.07). We also examined whether crows performed the same first action for the matched and the control stem; this was only possible for five subjects, since one bird did not manufacture a tool from the matched stem and one bird pulled the entire control stem out of the material log.

### Complementary analyses

Our results suggested that some of the variation observed in crows’ tool manufacture behaviour (Experiment 1) could be explained by the properties of raw materials (Experiment 2). Although we had attempted to collect stems of fairly standardised properties for Experiment 1, we noticed seasonal changes both in the properties of stems we could find in our study site, and in the crows’ manufacture behaviour (Fig. 5a). We had not recorded the diameter of stems before providing them to crows in Experiment 1, but we were able to gauge seasonal patterns by measuring the diameter of manufactured tools (Fig. 5b), which were routinely photographed on grid paper after trials. Using ImageJ software, one of us (SS) measured the diameter (*ca.* 1 cm above the joint on the tool shaft) of a subsample of 103 hooked stick tools manufactured by 16 subjects across 19 trials of Experiment 1 (deployment could not be confirmed for all of them). Trials were selected pseudorandomly for analysis, in a way that achieved even coverage of our study period at approximately one-week intervals.

We used (G)LMMs, with bird ID and year fitted as random effects, to analyse seasonal changes (trial date) in: (i) stem diameter; and (ii) time to release the basic tool (both log-transformed to normalise errors). Given the observed quadratic relationship between plant properties and the time taken to release the basic tool in Experiment 2 (see above), we also fitted models with a quadratic date term, yielding lower AICs compared to those without this term (for [i]: −38.2 vs. –34.5, *P =* 0.02; for [ii]: 188.2 vs. 199.9, *P =* 0.01). As some birds were tested sequentially, we removed bird ID as a random effect for analysing temporal changes in the number of actions in sequences; for this test, we constructed GLMs with a Poisson error structure and log link function, only using the first three sequences from each subject, to avoid overrepresentation of subjects with more sequences. Significance of the main effect was tested using the ‘lmtest’ package in R [69]. We also examined whether behavioural sequences changed over time (Experiment 1), and varied across material scores (Experiment 2), by testing for correlations between NW distance and time interval between trial dates, or differences in material scores, respectively. NW distance was calculated as described above, except that the sequences contained incomplete actions for these analyses (see Fig. 5a).

## Additional files

Additional file 1: Movie 1. Behavioural actions of hooked stick tool manufacture – Release of a basic tool. Scenes 1–3, release of the basic tool by cutting. Scenes 4–6, release of the basic tool by pulling. (MP4 19555 kb) Additional file 2: Table S1. Behavioural actions during hooked stick tool manufacture. (PDF 203 kb) Additional file 3: Movie 2. Behavioural actions of hooked stick tool manufacture – Modifications. Scene 1, processing the tool by stripping bark. Methods of bending the tool shaft: Scene 2, trapping the tool under the foot and bending the tool shaft with the bill; Scene 3, holding the tool in the bill and pressing the tool shaft against a firm substrate; and Scene 4, inserting the tool into a hole and pulling the tool shaft sideways by bill. (MP4 20519 kb) Additional file 4: Movie 5. Complete manufacture sequence by an adult crow. (MP4 20212 kb) Additional file 5: Movie 4. Complete manufacture sequence by an immature crow. (MP4 19000 kb) Additional file 6: Figure S1. Material and manufacture sequences of hooked stick tools in Experiment 2. (a) Photographs of sample stems for material scores 1, 4/5 and 8. The grey scale bars are 5 mm. (b) Manufacture sequences grouped by material score of the provided plant stems. Within each material score, sequences are ordered by individual as follows: AN1, CR8, EU7, HA7, EV0, AM7 and CE0. For a description of colour-coding and manufacture stages, see Fig. 2 in the main text. (PDF 135 kb) Additional file 7: Figure S2. Data on raw-material properties and tool-handling behaviour from two experiments. (a) In Experiment 1, the release of basic tools from plant material was fastest around 7 October, which corresponds to (b) a stem diameter of about 1.4 mm. (c) In Experiment 2, the diameter of stems increased with ‘material score’, as intended (tool-shaft diameter had been measured to the nearest 0.01 mm using digital calipers, approximately 1 cm from the joint), and a diameter of about 1.4 mm corresponded to (d) the fastest observed release of a basic tool. This provides evidence for an ‘optimal’ stem diameter for tool manufacture. Similar patterns were found for both when the same crow was simultaneously presented with stems of varying diameter (Experiment 2) and when different crows were (sequentially) provided with changing plant materials over the course of several months (Experiment 1). Note that panels (a), (b) and (c) are components of main text figures (Fig. 5c, b and 3c). (PDF 76 kb) Additional file 8: Movie 3. Complete manufacture sequence by a juvenile crow. (MP4 19373 kb)

## Footnotes

1 Assessment of inter-observer agreement: SS scored all valid manufacture sequences from Experiments 1 and 2, and BK re-scored a random sample of 12 (10 %) of them. SS and BK agreed on the first behavioural action in 11 of the 12 sequences (Cohen’s Kappa, *κ* = 0.83), which is very good considering the complexity of the behaviours investigated. Observers produced very similar estimates for the number of actions within sequences, both for the number of complete actions only (correlation coefficient, *r* = 0.93, *P* <0.001) and the number of actions including incomplete ones (*r* = 0.87, *P* <0.001). Out of the 12 sequences, SS and BK completely agreed on the number of actions in seven sequences, and among these, there were only two cases where the identities of behavioural actions differed between observers (‘bend’ and ‘process hook’; ‘pull’ and ‘sever hook’); all other disagreements were due to differences in the number of actions scored. Analyses reported in the main text and figures are based on the original scores. All sequences from Experiment 2 until the release of the basic tool were scored by BK and reviewed by SS. For the five trials in Experiment 2 where birds manufactured tools from both matched stems (ten valid manufacture sequences), BK and SS reached 100 % agreement on their independent scores for the first action (both place of grip and place of detachment). For any initial disagreement, both observers subsequently agreed on consensus classifications. In Experiment 2, inter-observer agreement for assigning ‘material scores’ to plant stems was excellent: for all 56 stems, at least two of the three observers agreed, and for 66 % of them, all three agreed; taking into account that disagreement on material scores 4 and 5 was irrelevant, as these two stems were experimentally matched (see Methods), all three observers agreed in 73 % of all cases.

## References

[1] Hunt GR (1996). Manufacture and use of hook-tools by New Caledonian crows. Nature.

[2] Rutz C, St Clair JJH (2012). The evolutionary origins and ecological context of tool use in New Caledonian crows. Behav Processes.

[3] Tebbich S, Teschke I, Cartmill EA, Stankewitz S (2012). Use of a barbed tool by an adult and a juvenile woodpecker finch (*Cactospiza pallida*). Behav Processes.

[4] Hunt GR, Gray RD (2004). The crafting of hook tools by wild New Caledonian crows. Proc R Soc B Biol Sci.

[5] Shumaker RW, Walkup KR, Beck BB (2011). Animal tool behavior: The use and manufacture of tools by animals.

[6] McBrearty S, Brooks AS (2000). The revolution that wasn’t: A new interpretation of the origin of modern human behavior. J Hum Evol.

[7] Yellen JE, Brooks AS, Cornelissen E, Mehlman MJ, Stewart K (1995). A middle stone age worked bone industry from Katanda, Upper Semliki Valley, Zaire. Science.

[8] O’Connor S, Ono R, Clarkson C (2011). Pelagic fishing at 42,000 years before the present and the maritime skills of modern humans. Science.

[9] Gramsch B, Beran J, Hanik S, Sommer RS (2013). A palaeolithic fishhook made of ivory and the earliest fishhook tradition in Europe. J Archaeol Sci.

[10] Barnes B, Edwards B, Hallam J, Stuart A (1971). Skeleton of a Late Glacial elk associated with barbed points from Poulton-le-Fylde, Lancashire. Nature.

[11] Churchill SE (1993). Weapon technology, prey size selection, and hunting methods in modern hunter-gatherers: Implications for hunting in the Palaeolithic and Mesolithic. Archeological Pap Am Anthropol Assoc.

[12] Laland KN, Galef BG (2009). The question of animal culture.

[13] Whiten A, Goodall J, McGrew WC, Nishida T, Reynolds V, Sugiyama Y (1999). Cultures in chimpanzees. Nature.

[14] Rendell L, Whitehead H (2001). Culture in whales and dolphins. Behav Brain Sci.

[15] van Schaik CP, Ancrenaz M, Borgen G, Galdikas B, Knott CD, Singleton I (2003). Orangutan cultures and the evolution of material culture. Science.

[16] Krützen M, Mann J, Heithaus MR, Connor RC, Bejder L, Sherwin WB (2005). Cultural transmission of tool use in bottlenose dolphins. Proc Natl Acad Sci U S A.

[17] Wich SA, Krützen M, Lameira AR, Nater A, Arora N, Bastian ML (2012). Call cultures in orang-utans?. PLoS One.

[18] Laland KN, Janik VM (2006). The animal cultures debate. Trends Ecol Evol.

[19] Meulman EJM, Seed AM, Mann J (2013). If at first you don’t succeed… Studies of ontogeny shed light on the cognitive demands of habitual tool use. Phil Trans R Soc B Biol Sci.

[20] Koops K, Visalberghi E, van Schaik CP (2014). The ecology of primate material culture. Biol Lett.

[21] Tebbich S, Taborsky M, Fessl B, Blomqvist D (2001). Do woodpecker finches acquire tool-use by social learning?. Proc R Soc B Biol Sci.

[22] Biro D, Inoue-Nakamura N, Tonooka R, Yamakoshi G, Sousa C, Matsuzawa T (2003). Cultural innovation and transmission of tool use in wild chimpanzees: Evidence from field experiments. Anim Cogn.

[23] Kenward B, Weir AAS, Rutz C, Kacelnik A (2005). Tool manufacture by naive juvenile crows. Nature.

[24] Lonsdorf EV, Hopkins WD (2005). Wild chimpanzees show population-level handedness for tool use. Proc Natl Acad Sci U S A.

[25] Lonsdorf E (2006). What is the role of mothers in the acquisition of termite-fishing behaviors in wild chimpanzees (*Pan troglodytes schweinfurthii*)?. Anim Cogn.

[26] De Resende BD, Ottoni EB, Fragaszy DM (2008). Ontogeny of manipulative behavior and nut-cracking in young tufted capuchin monkeys (*Cebus apella*): A perception–action perspective. Dev Sci.

[27] Hopkins WD, Reamer L, Mareno MC, Schapiro SJ (2015). Genetic basis in motor skill and hand preference for tool use in chimpanzees (*Pan troglodytes*). Proc R Soc B Biol Sci.

[28] Humle T, Matsuzawa T (2002). Ant-dipping among the chimpanzees of Bossou, Guinea, and some comparisons with other sites. Am J Primatol.

[29] Fox EA, van Schaik CP, Sitompul A, Wright DN (2004). Intra- and interpopulational differences in orangutan (*Pongo pygmaeus*) activity and diet: Implications for the invention of tool use. Am J Phys Anthropol.

[30] Sanz CM, Morgan DB (2013). Ecological and social correlates of chimpanzee tool use. Phil Trans R Soc B Biol Sci.

[31] Möbius Y, Boesch C, Koops K, Matsuzawa T, Humle T (2008). Cultural differences in army ant predation by West African chimpanzees? A comparative study of microecological variables. Anim Behav.

[32] Richerson PJ, Boyd R (2005). Not by genes alone: How culture transformed human evolution.

[33] Hunt GR, Gray RD (2003). Diversification and cumulative evolution in New Caledonian crow tool manufacture. Proc R Soc B Biol Sci.

[34] Hunt GR, Gray RD (2006). Tool manufacture by New Caledonian crows: Chipping away at human uniqueness. Acta Zool Sin.

[35] Bluff LA, Kacelnik A, Rutz C (2010). Vocal culture in New Caledonian crows *Corvus moneduloides*. Biol J Linn Soc.

[36] Hansell MH (2005). Animal architecture.

[37] Hansell M, Ruxton GD (2008). Setting tool use within the context of animal construction behaviour. Trends Ecol Evol.

[38] Andrefsky W (1994). Raw-material availability and the organization of technology. Am Antiquity.

[39] Eren MI, Roos CI, Story BA, von Cramon-Taubadel N, Lycett SJ (2014). The role of raw material differences in stone tool shape variation: An experimental assessment. J Archaeol Sci.

[40] Gaino E, Cianficconi F, Rebora M, Todini B (2002). Case-building of some Trichoptera larvae in experimental conditions: Selectivity for calcareous and siliceous grains. Italian J Zool.

[41] Bailey IE, Morgan KV, Bertin M, Meddle SL, Healy SD (2014). Physical cognition: Birds learn the structural efficacy of nest material. Proc R Soc B Biol Sci.

[42] St Clair JJH, Klump BC, van der Wal JEM, Sugasawa S, Rutz C. Strong between-site variation in New Caledonian crows’ use of hook-tool-making materials. Biol J Linn Soc. In press. 10.1111/bij.12757 PMC5111415 27867222

[43] St Clair JJH, Rutz C (2013). New Caledonian crows attend to multiple functional properties of complex tools. Phil Trans R Soc B Biol Sci.

[44] Wasserman L (2011). All of statistics.

[45] Boyd R, Richerson PJ (2004). The origin and evolution of cultures.

[46] Sanz C, Call J, Morgan D (2009). Design complexity in termite-fishing tools of chimpanzees (*Pan troglodytes*). Biol Lett.

[47] Troscianko J, von Bayern AM, Chappell J, Rutz C, Martin GR (2012). Extreme binocular vision and a straight bill facilitate tool use in New Caledonian crows. Nat Commun.

[48] Bluff LA, Troscianko J, Weir AAS, Kacelnik A, Rutz C (2010). Tool use by wild New Caledonian crows *Corvus moneduloides* at natural foraging sites. Proc R Soc B Biol Sci.

[49] Rutz C, Bluff LA, Reed N, Troscianko J, Newton J, Inger R (2010). The ecological significance of tool use in New Caledonian crows. Science.

[50] Hunt GR, Holzhaider JC, Gray RD (2012). Prolonged parental feeding in tool-using New Caledonian crows. Ethology.

[51] Kenward B, Rutz C, Weir AAS, Kacelnik A (2006). Development of tool use in New Caledonian crows: Inherited action patterns and social influences. Anim Behav.

[52] Koops K, Schöning C, Isaj M, Hashimoto C (2015). Cultural differences in ant-dipping tool length in neigbouring communities of chimpanzees at Kalinzu, Uganda. Sci Rep.

[53] Walsh PT, Hansell M, Borello WD, Healy SD (2010). Repeatability of nest morphology in African weaver birds. Biol Lett.

[54] Fragaszy DM, Greenberg R, Visalberghi E, Ottoni EB, Izar P, Liu Q (2010). How wild bearded capuchin monkeys select stones and nuts to minimize the number of strikes per nut cracked. Anim Behav.

[55] Brownlow A, Plumptre A, Reynolds V, Ward R (2001). Sources of variation in the nesting behavior of chimpanzees (*Pan troglodytes schweinfurthii*) in the Budongo Forest, Uganda. Am J Primatol.

[56] Sanz C, Morgan D, Gulick S (2004). New insights into chimpanzees, tools, and termites from the Congo Basin. Am Naturalist.

[57] Pascual-Garrido A, Buba U, Nodza G, Sommer V (2012). Obtaining raw material: Plants as tool sources for Nigerian chimpanzees. Folia Primatol.

[58] van Casteren A, Sellers WI, Thorpe SK, Coward S, Crompton RH, Myatt JP (2012). Nest-building orangutans demonstrate engineering know-how to produce safe, comfortable beds. Proc Natl Acad Sci U S A.

[59] Amick DS, Mauldin RP (1997). Effects of raw material on flake breakage patterns. Lithic Technol.

[60] Sanz C, Call J, Boesch C (2013). Tool use in animals: Cognition and ecology.

[61] Kenward B, Rutz C, Weir AAS, Chappell J, Kacelnik A (2004). Morphology and sexual dimorphism of the New Caledonian crow *Corvus moneduloides*, with notes on its behaviour and ecology. Ibis.

[62] Heinrich B, Marzluff J (1992). Age and mouth color in common ravens. Condor.

[63] Klump BC, van der Wal JEM, St Clair JJH, Rutz C (2015). Context-dependent ‘safekeeping’of foraging tools in New Caledonian crows. Proc R Soc B Biol Sci.

[64] Bates D, Maechler M, Bolker B, Walker S. Lme4: Linear mixed-effects models using Eigen and S4. R package version 11–6; 2014.

[65] R Core Team. R: A language and environment for statistical computing. Vienna: R Foundation for Statistical Computing; 2014.

[66] Needleman SB, Wunsch CD (1970). A general method applicable to the search for similarities in the amino acid sequence of two proteins. J Mol Biol.

[67] Pages H, Aboyoun P, Gentleman R, DebRoy S. Biostrings: String objects representing biological sequences, and matching algorithms. R package version 2.36.1; 2015.

[68] Oksanen J, Blanchet F, Kindt R, Legendre P, Minchin P, O’Hara R, et al. vegan: Community Ecology Package. R package version 2.2-1; 2015.

[69] Zeileis A, Hothorn T (2002). Diagnostic checking in regression relationships. R News.

