# Supplementary material for: Hook tool manufacture in New Caledonian crows: behavioural variation and the influence of raw materials
Source: BMC Biol. 2015 Nov 18;13:97. doi: 10.1186/s12915-015-0204-7 (PMC4650250; doi:10.1186/s12915-015-0204-7)
Supplement: Additional file 2: Table S1. — Behavioural actions during hooked stick tool manufacture. (PDF 203 kb) [file 12915_2015_204_MOESM2_ESM.pdf]

Supplementary Table 1 Behavioural actions during hooked stick tool manufacture.

Stage	Action type	Coding	Definition
Release of basic tool	Cut	Event	Detachment happens at the point of grip.
	Pull	Event	Detachment happens away from the point of grip.
Actions completed through multiple attempts / incomplete attempt	Incomplete attempt	Event	The subject tries an action and fails to complete it.
	Most-effective attempt	Event	An action is completed through multiple attempts and this is the most-effective attempt (not the last, see below).
	Last attempt	Event	An action is completed through multiple attempts and this is the last attempt (not the most-effective, see below).
	Most-effective and last attempt	Event	An action is completed through multiple attempts, and the most-effective attempt is the last attempt.
Processing of basic tool	Sever hook shaft	Event	The subject shortens the hook shaft by severing the stem instantaneously.
	Sever tool shaft	Event	The subject shortens the tool shaft by severing the stem instantaneously.
	Sever root shaft	Event	The subject shortens the root shaft by severing the stem instantaneously.
	Nibble tool shaft	State	The subject processes the tool shaft by nibbling the end of it. This does not involve severing the stem instantaneously.
	Process hook	State	The subject crafts the hook of the basic tool or tool by nibbling it or peeling bark from it.
	Bend tool shaft	State	The subject actively deforms the tool shaft of the basic tool or tool. This action is further classified based on how the subject carries it out (see main text).
Deployment of tool	Insert tool	Event	The subject holds the tool in its bill and inserts it into a hole. This is only scored when the subject first inserts the tool.
End of scoring	Successful	Event	The subject extracts bait.
	Abandoned	Event	The subject abandons the tool for at least two minutes.
	Time-out	Event	The subject has neither extracted bait nor abandoned the tool five minutes after the first insertion.
