# Supplementary figures and images for: Hook tool manufacture in New Caledonian crows: behavioural variation and the influence of raw materials
Source: BMC Biol. 2015 Nov 18;13:97. doi: 10.1186/s12915-015-0204-7 (PMC4650250; doi:10.1186/s12915-015-0204-7)

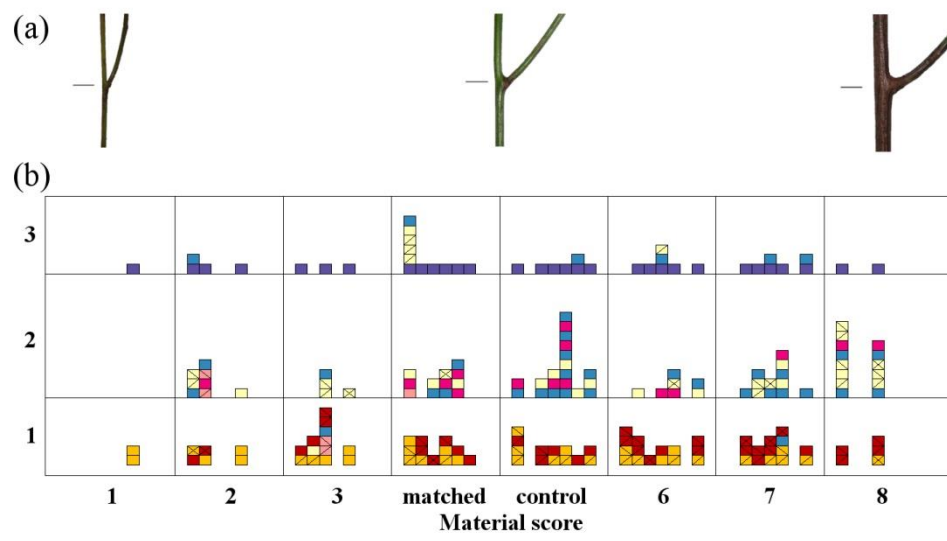

Supplement: Additional file 6: Figure S1. — Material and manufacture sequences of hooked stick tools in Experiment 2. (a) Photographs of sample stems for material scores 1, 4/5 and 8. The grey scale bars are 5 mm. (b) Manufacture sequences grouped by material score of the provided plant stems. Within each material score, sequences are ordered by individual as follows: AN1, CR8, EU7, HA7, EV0, AM7 and CE0. For a description of colour-coding and manufacture stages, see Fig. 2 in the main text. (PDF 135 kb) [file 12915_2015_204_MOESM6_ESM.pdf]

## Experiment 1

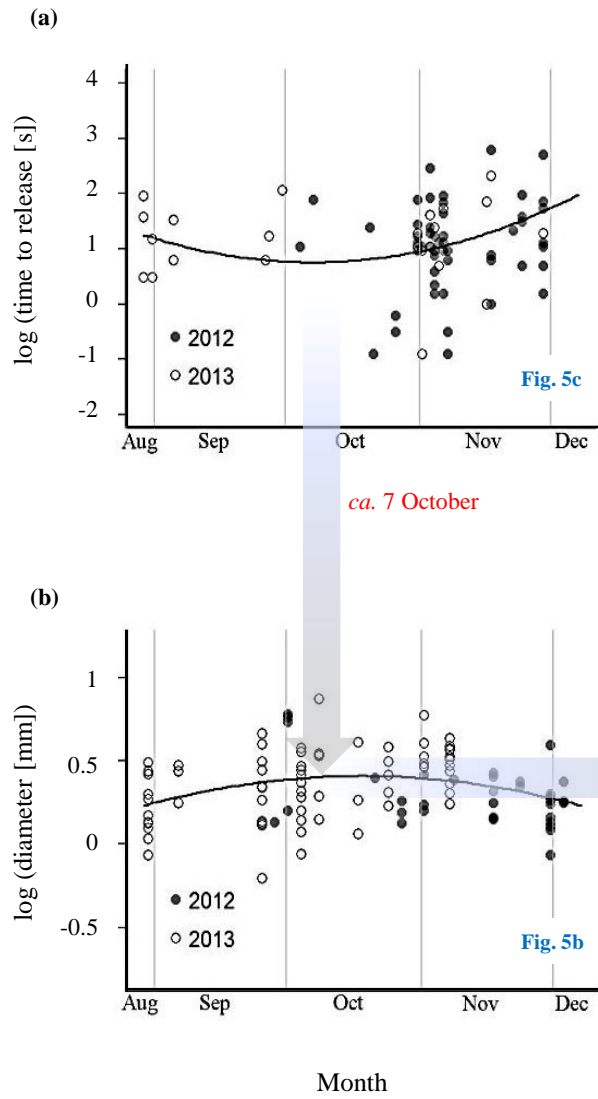

## Experiment 2

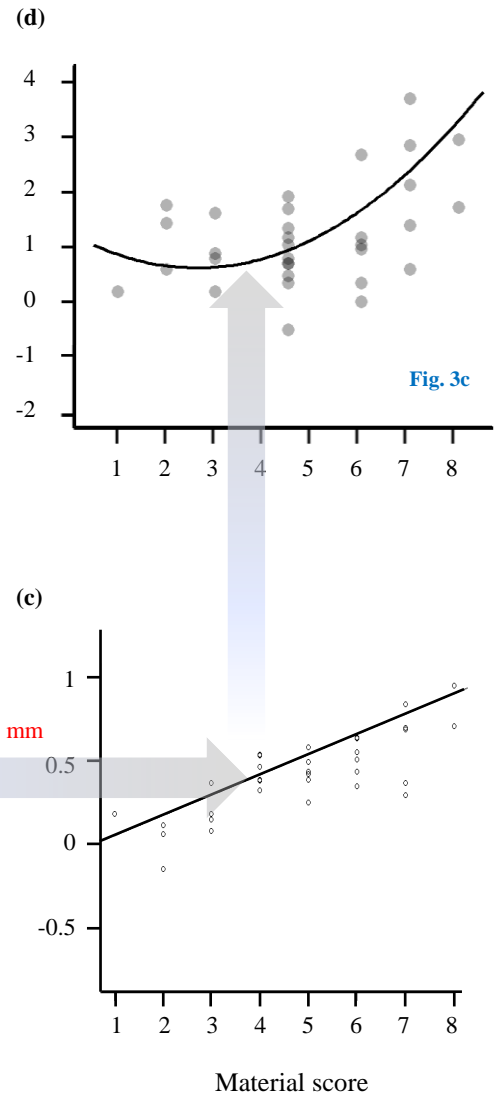

Supplement: Additional file 7: Figure S2. — Data on raw-material properties and tool-handling behaviour from two experiments. (a) In Experiment 1, the release of basic tools from plant material was fastest around 7 October, which corresponds to (b) a stem diameter of about 1.4 mm. (c) In Experiment 2, the diameter of stems increased with ‘material score’, as intended (tool-shaft diameter had been measured to the nearest 0.01 mm using digital calipers, approximately 1 cm from the joint), and a diameter of about 1.4 mm corresponded to (d) the fastest observed release of a basic tool. This provides evidence for an ‘optimal’ stem diameter for tool manufacture. Similar patterns were found for both when the same crow was simultaneously presented with stems of varying diameter (Experiment 2) and when different crows were (sequentially) provided with changing plant materials over the course of several months (Experiment 1). Note that panels (a), (b) and (c) are components of main text figures (Fig. 5c, b and 3c). (PDF 76 kb) [file 12915_2015_204_MOESM7_ESM.pdf]
